# Outcomes of rigid fixation compared to wire closure after median sternotomy a systematic review and meta-analysis

**DOI:** 10.1097/MS9.0000000000003259

**Published:** 2025-04-04

**Authors:** Oshan Shrestha, Sujan Bohara, Suchit Thapa Chettri, Ashim Pandey, Utshab Acharya, Ashish Tiwari, Nikesh Bhandari, Prashiddha Bikram Kadel, Kajan Raj Shrestha

**Affiliations:** a Department of Cardiothoracic and Vascular Surgery, Manmohan Cardiothoracic Vascular and Transplant Center, Kathmandu, Nepal; b Department of Cardiovascular Surgery, Shahid Gangalal National Heart Centre, Kathmandu, Nepal; c College of Medicine, Nepalese Army Institute of Health Sciences, Kathmandu, Nepal; d Department of Cardiothoracic and Vascular Anaesthesiology, Manmohan Cardiothoracic Vascular and Transplant Center, Kathmandu, Nepal

**Keywords:** rigid fixation, sternotomy, wire cerclage

## Abstract

**Introduction:**

Wire closure is linear and provides a single point of support, while rigid fixation provides two-dimensional support and acts as an additional skeletal brace that holds the two hemisternum together. This property avoids the factors that make the wire closure unstable. This study aims to compare the outcomes of rigid fixation with wire closure to study the difference in recovery time, pain scores, and sternal complications.

**Methods:**

Prospective protocol registration was done, and electronic databases were searched without using any search filters. Screening was performed by independent reviewers, and data was extracted from selected studies. Heterogeneity was assessed by the *I*^2^ test, the effect model was chosen accordingly, and the effect measure was chosen as appropriate. Forest plots and funnel plots were used to give visual feedback.

**Results:**

The rigid fixation group had better healing scores at 3 months (MD: 0.8; 95% CI: 0.59–1.01; *n* = 376; *I*^2^ = 0%; *P* = <0.00001) and at 6 months (MD: 0.71; 95% CI: 0.23–1.20; *n* = 376; *I*^2^ = 71%; *P* = 0.004). The rigid fixation group had a better pain score at 3 months and had a lesser incidence of sternal dehiscence. However, rigid fixation group took 2.91 minutes longer for closure and had a 1.02-day shorter hospital stay on average.

**Conclusion:**

Rigid fixation was found to be superior to the wire cerclage in regard to shorter hospital stay duration, sternal healing scores, postoperative pain up to 3 months, and sternal dehiscence complication among the obese population.

## Introduction

After the introduction of cardiopulmonary bypass machine and cardioplegic solutions in the 1950s, complex cardiothoracic surgeries became possible, and the median sternotomy approach gained popularity among cardiothoracic surgeons^[1]^. Median sternotomy has been used widely by cardiothoracic surgeons since then, and its closure has been performed conventionally by using stainless steel wires. Although the stainless-steel wire closure is able to achieve sternal edge approximation, the wires become loose over time, and stresses concentrated at the wires cause it to cut through the bone and allow motion of varying degrees. Also, osteoporosis, coughing, and chest movements contribute to the instability. This can lead to separation, pain, delay in recovery, and sternal complications^[2,3]^.

**Highlights**

- Wire closure provides a single point of support, while rigid fixation provides two-dimensional support.
- Rigid fixation group had better healing scores at 3 and 6 months.
- Rigid fixation group had a better pain score at 3 months and had a lesser incidence of sternal dehiscence.
- Rigid fixation group took 2.91 minutes longer for closure and had a 1.02-day shorter hospital stay on average.

The method of rigid fixation using plates and screws for sternal closure has come into practice and has been started to be used over the years as it provides two-dimensional support and acts as an additional skeletal brace holding the two hemisternum together, unlike wire closure^[4]^. This property is thought to avoid the factors that render the wire closure unstable, and various biomechanical and clinical studies have shown the benefits of rigid fixation^[4,5]^.

This systematic review and meta-analysis aim to compare the outcomes of rigid fixation with wire closure to study the difference in recovery time, pain scores, sternal complications, and cost.

## Materials and methods

This study is in the line with the PRISMA guidelines^[6]^ and the AMSTAR guidelines^[7]^.

### Protocol registration

The study protocol was registered prospectively in the International Prospective Register of Systematic Reviews (PROSPERO) and in the Registry of Systematic Reviews/Meta-Analyses.

### Search strategy

Electronic databases (PubMed, PubMed Central, Embase, and Scopus) were searched in this study by using search terms like (“rigid fixation”), (“plate fixation”), (“wire cerclage”), (“wire closure”), (sternum), and (sternotomy) with appropriate Boolean operators. No search filters were applied during the search, and there were no time restrictions in the search. The details of the search strategy and search results are available in Supplementary File 1, http://links.lww.com/MS9/A787.

### Inclusion criteria and exclusion criteria

All of the comparative studies (randomized clinical trial, non-randomized trial, and cohort studies) that compared the outcomes of rigid fixation and wire closure used for median sternotomy closure were included in this study. Non-comparative studies, case reports, case series, viewpoints, and editorials were not part of this study.

### Study selection

The search results obtained from the four databases were imported to the Covidence software^[8]^ for screening. During the screening process, three independent reviewers screened the articles. Two reviewers screened the studies, and any conflict that arose was resolved by the third reviewer. The roles of the reviewers were exchanged during the title-and-abstract screening and the full-text screening phases.

### Data curation

The articles that matched the inclusion criteria were moved onto the data extraction phase. A template was prepared in Word with headings like study details, population, intervention, and comparator, and the template was used during the data collection. The author’s name, year of publication, demographic details of the participants, baseline characteristics of the population, the intervention of the study, the comparator of the study, and the outcomes such as time taken for sternal closure, duration of intensive care unit stay, duration of hospital stay, pain scores, sternal complications, and mortality were extracted.

### Data synthesis

The categorical variables were studied by using the odds ratio, and the continuous variables were studied with the mean difference/standardized mean difference. The level of heterogeneity was assessed by the *I*^2^ test, and the effect model for the analyses was chosen as per the heterogeneity level^[9]^. For heterogeneity up to 40%, the fixed effect model was used, and beyond this, the random effect model was used during the analyses. The results were expressed with 95% confidence interval, and the forest plots were used for visual feedback. The subgroup analysis was performed for an outcome by including randomized studies only and for outcomes reported for obese patients.

### Risk of bias assessment

Three independent reviewers were involved in the critical appraisal of the studies. The risk of bias (ROB) tool was used for randomized studies, and the Risk of Bias in Non-randomized Studies – of Interventions (ROBINS-I) was used for non-randomized studies to assess the risk of bias among the included studies. The assessment of bias is shown in Figures A and B of Supplementary File 2, http://links.lww.com/MS9/A788.

### Sensitivity analysis and publication bias

The sensitivity analysis was carried out by excluding each study at a time to observe if it made any significant changes in the analysis and its result. The publication bias was assessed with the help of a funnel plot for the analyses, which included at least ten studies^[9]^.

## Results

**Figure 1.**
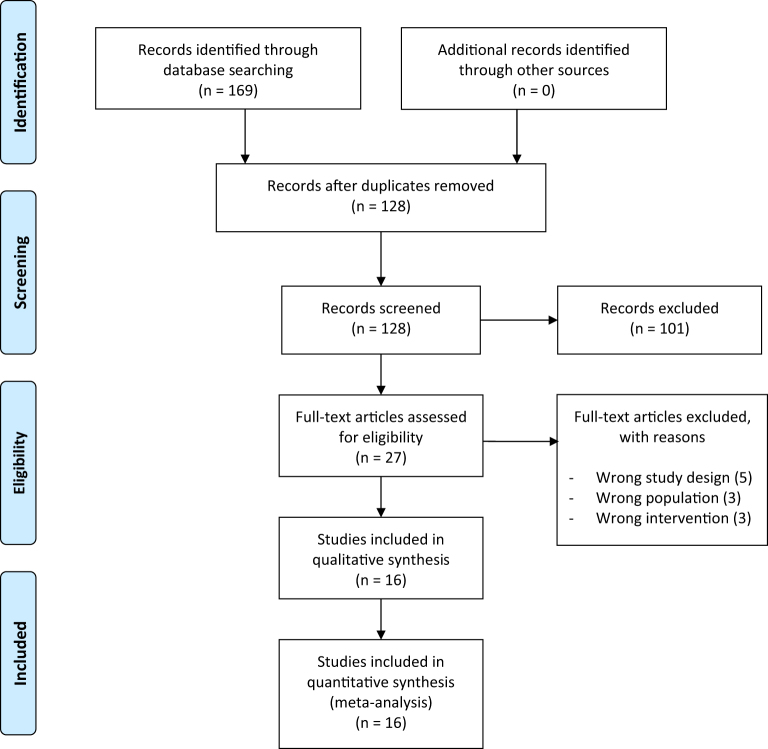
PRISMA flow chart.

This systematic review and meta-analysis of 14 comparative studies (results of which are published in a total of 16 articles) involved a total population of 2452 patients. Out of 14 comparative studies that were included, 4 were randomized studies, and others were non-randomized comparative studies. The search of four databases yielded a total of 169 studies, and after the removal of the duplicates, 128 studies were left for screening. After the screening, 16 studies were identified as a match to the inclusion criteria of the study. Details of screening are shown in Fig. 1.

### Qualitative synthesis

This study included a total of 14 comparative studies that were published from 2009 to 2023. Out of 14 studies, four studies included obese population only, while others included participants indiscriminately of body weight. Details of the included 14 comparative studies (16 publications) in the Population, Intervention, Comparator, and Outcome (PICO) format: under population (P), the total number of participants in each arm is listed along with their baseline characteristics; the surgical procedure under study is given under intervention (I); the procedure with which the intervention is compared is under comparator (C); and the variables of interest are kept under outcomes (O). The details are shown in Table 1.

**Table 1.** Details of the included studies

Study no	Publication no	Study details	Population (N)	Intervention (T)	Comparator (C)	Outcomes
1	1	Allen *et al*, 2016 ^[10]^ Randomized Controlled Trial	N= 236 (T= 116, C= 120)Male T= 86/116, C= 91/120Female T= 30/116, C= 29/120**Value (Mean ± SD)**Age (years): T= 65.3 ± 13.0, C= 65.7 ± 11.4Height (cm): T= 172.2 ± 9.8, C= 172.7 ± 9.9Weight (kg): T= 85.6 ± 17.6, C= 88.2 ± 16.5Body Mass Index (kg/m^2^): T= 28.8 ± 4.7, C= 29.4 ± 4.6**Comorbidities**Hypertension: T= 86/116, C= 83/120Peripheral arterial disease: T= 12/116, C= 5/120Cerebrovascular disease: T= 10/116, C= 7/120Diabetes: T= 35/116, C= 44/120Current tobacco use: T= 14/116, C= 10/120Chronic lung disease: T= 22/116, C= 22/120**Intraoperative variables**Isolated CABG: T= 56/116, C= 57/120Isolated valve: T= 33/116, C= 33/120CABG/valve: T= 25/116, C= 28/120	Rigid sternal fixation	Wire fixation	**Value (Mean ± SD)**Duration of procedure (hours): T= 5.6 ± 1.8, C= 5.6 ± 1.4Sternal closure time (minutes): T= 18.9 ± 9.0, C= 16.3 ± 9.3Duration of hospital stay (days): T= 6.9 ± 2.4, C= 6.9 ± 2.7Index hospitalization cost: T= 23437 ± 112421, C= 20574 ± 14102**At 1 month**Mortality: T= 2/116, C= 2/120Sternal wound infection: T= 0, C= 3/120**At 3 months**Sternal healing score: T= 2.6 ± 1.1, C= 1.8 ± 1.0Total cost at 3 months: T= 29179 ± 21016, C= 31067 ± 28562Sternal union: T= 42/103, C= 16/102Sternal complications: T= 0, C= 5/120**At 6 months**Sternal healing score: T= 3.8 ± 1.0, C= 3.3 ± 1.1Total cost at 6 months: T= 32439 ± 24124, C= 34085 ± 30916Sternal union: T= 81/101, C= 67/100Sternal complications: T= 0, C= 6/120Sternal wound infection: T= 0, C= 5/120**Resting pain score**(0 representing no pain and 10 representing the worst pain)1 week: T= 1.1 ± 0.18 (0.5-1.6), C= 2.1 ± 0.36 weeks: T= 0.6 ± 0.08, C= 1.2 ± 0.1333 months: T= 0.5 ± 0.1, C= 0.6 ± 0.0676 months: T= 0.6 ± 0.083, C= 1.2 ± 0.133**Coughing pain score**(0 representing no pain and 10 representing the worst pain)1 week: T= 2.2 ± 0.50, C= 4.1 ± 0.586 weeks: T= 1.35 ± 0.30, C= 1.9 ± 0.263 months: T= 0.8 ± 0.30, C= 0.9 ± 0.306 months: T= 0.5 ± 0.33, C= 0.6 ± 0.33
	2	Allen *et al*, 2018 ^[11]^ Randomized Controlled Trial				
	3	Allen *et al*, 2018 ^[12]^ Randomized Controlled Trial				
2	4	Atik *et al*, 2023 ^[13]^ Case-control study Obese study population	N= 368 (T= 121, C= 247)Male T= 30/121, C= 61/247Female T= 91/121, C= 186/247**Value (Mean ± SD)**Age (years): T= 62 ± 5.16, C= 59 ± 6.72Body Mass Index (kg/m^2^): T= 41.1 ± 2.1, C= 40.9 ± 1.9Ejection fraction: T= 51 ± 10.1, C= 50 ± 8.8**Comorbidities**Diabetes mellitus: T= 112/121, C= 218/247Peripheral vascular disease: T= 5/121, C= 8/247Hypertension: T= 98/121, C= 193/247COPD: T= 34/121, C= 59/247**Intraoperative variables**Isolated CABG: T= 88/121, C= 182/247Isolated AVR: T= 7/116, C= 13/120Isolated MVR: T= 6/116, C= 14/120AVR + MVR: T= 3/116, C= 7/120CABG + AVR: T= 10/116, C= 21/120CABG + MVR: T= 7/116, C= 10/120	Rigid sternal fixation + Wire fixation	Wire fixation	**Value (Mean ± SD)**Cardiopulmonary bypass time (minutes): T= 138 ± 51.21, C= 133 ± 41.55Duration of procedure (hours): T= 5.75 ± 1.04, C= 5.03 ± 0.83**Complications**Wound drainage: T= 2/121, C= 18/247Sternal dehiscence: T= 3/121, C= 28/247Mediastinitis: T= 0, C= 4/247Sternal revision: T= 1/121, C= 22/247**At 30 days**Mortality: T= 4/121, C= 7/247
3	5	Celik *et al*, 2022 ^[14]^ Retrospective study Obese study population	N= 124 (T= 36, C= 88)Male T= 10/36, C= 61/88Female T= 26/36, C= 27/88**Value [Median (IQR)]**Age (years): T= 64 (59-70), C= 63 (55-69)Ejection fraction: T= 57 (50-65), C= 55 (45-63.7)Euro Score: T= 2.03 (1.33-2.63), C= 1.35 (0.83-2.10)Body Mass Index (kg/m^2^)≥30-34.99: T= 16/36, C= 72/88≥35-39.99: T= 14/36, C= 13/88≥40: T= 6/36, C= 3/88**Comorbidities**Diabetes mellitus: T= 33/36, C= 44/88COPD: T= 17/36, C= 27/88**Intraoperative variables**CABG: T= 30/36, C= 74/88Off-pump CABG: T= 2/36, C= 6/88AVR: T= 1/36, C= 5/88MVR: T= 3/36, C= 3/88	Rigid sternal fixation + Wire fixation	Wire fixation	**Value (Mean ± SD)**Cardiopulmonary bypass time (minutes): T= 118 ± 13.25, C= 119 ± 7.33Duration of hospital stay (days): T= 8 ± 1, C= 8 ± 0.5Duration of Intensive care unit stay (hours): T= 72 ± 10.32, C= 72 ± 7.92Duration of ventilator use (hours): T= 5 ± 0.812, C= 4.5 ± 0.33**Complications**Mortality: T= 3/36, C= 4/88Sternal complications: T= 4/36, C= 8/88Sternal infection: T= 2/36, C= 4/88Non-infectious sternal complications: T= 2/36, C= 4/88Sternal dehiscence: T= 0, C= 3/88
4	6	Elghonemy *et al*, 2016 ^[15]^ Non-randomized trial	N= 30 (T= 15, C= 15)Male T= 11/15, C= 10/15Female T= 4/15, C= 5/15**Value (Mean ± SD)**Age (years): T= 57.07 ± 8.65, C= 59.27 ± 9.06Body Mass Index (kg/m^2^): T= 30.60 ± 3.54, C= 30.73 ± 3.45HbA1c: T=6.73 ± 0.76, C= 6.61 ± 0.93Renal function (Cr): T= 1.92 ± 1.38, C=1.97 ± 1.31**Comorbidities**Diabetes mellitus: T= 10/15, C= 9/15COPD: T= 6/15, C= 5/15Renal Function (Haemodialysis): T= 2/15, C= 3/15	Rigid sternal fixation	Wire fixation	**Value (Mean ± SD)**Duration of hospital stay (days): T= 7.27 ± 2.55, C= 8.80 ± 3.08Cardiopulmonary bypass time (minutes): T= 75.1 ± 14.3, C= 78.2 ± 13.1Duration of ventilator use (hours): T=8.0 ± 3.38, C= 7.73 ± 3.58**Pain scores**(0 representing no pain and 10 representing the worst pain)In hospital: T= 5.13 ± 1.25, C= 6.40 ± 0.50At 1 month: T= 2.53 ± 1.25, C= 3.93 ± 0.80At 6 months: T= 1.0 ± 0.93, C= 1.67 ± 1.11Narcotic use (milligram): T= 8.0 ± 4.71, C= 14 ± 5.73**Complications**Re-exploration: T= 0, C= 2/15Wound infection: T= 2/15, C= 3/15Refixation: T= 0, C= 1/15Sternal instabilityIn hospital: T= 0, C= 2/15At 1 month: T= 1/15, C= 1/15
5	7	Hirose *et al*, 2011 ^[4]^ Retrospective study	N= 222 (T= 89, C= 133)Male T= 75/89, C= 64/133Female T= 14/89, C= 69/133**Value (Mean ± SD)**Age (years): T= 62 ± 9, C= 69 ± 11Number of distal anastomoses in surgery: T= 2.6 ± 0.9, C= 2.5 ± 0.9**Cardiac profile**Acute myocardial infarction: T= 5/89, C= 5/133History of congestive heart failure: T= 11/89, C= 15/133Poor ejection function (<40%): T= 10/89, C= 20/133Atrial fibrillation: T= 6/89, C= 20/133Redo surgery: T= 2/89, C= 12/133Emergent or urgent surgery: T= 16/89, C= 35/133Left main disease: T= 15/89, C= 20/133**Comorbidities**Hypertension: T= 76/89, C= 117/133Diabetes: T= 27/89, C= 51/133Insulin user: T= 7/89, C= 20/133Hyperlipidemia: T= 75/89, C= 99/133Body mass index above 30: T= 18/89, C= 18/133Body mass index above 35: T= 50/89, C= 49/133Family history: T= 18/89, C= 24/133Peripheral vascular disease: T= 50/89, C= 63/133Cerebral vascular accident: T= 12/89, C= 13/133Chronic pulmonary disease: T= 11/89, C= 22/133Renal dysfunction (Creatinine above 1.5 mg/dl): T= 2/89, C= 6/133**Intraoperative variables**Coronary artery bypass surgery: T= 73/89, C= 84/133Valve surgery: T= 26/89, C= 72/133	Rigid sternal fixation	Wire fixation	**Value (Mean ± SD)**Duration of ventilator use (hours): T= 13 ± 20, C= 39 ± 97Duration of intensive care unit stay (hours): T= 58 ± 40, C= 99 ± 119Duration of hospital stay (days): T= 7.0 ± 3.7, C= 8.4 ± 4.7Re-exploration: T= 1/89, C= 0**Complications**Heart failure: T= 0/89, C= 0/133Postoperative myocardial infarction: T= 1/89, C= 1/133Pneumonia: T= 1/89, C= 5/133Cerebral vascular accident: T= 0/89, C= 5/133Sternal infection: T= 1/89, C= 0/133Total mortality: T= 1/89, C= 1/133
6	8	Liao *et al*, 2019 ^[16]^ Retrospective study Obese study population	N= 20 (T= 8, C= 14)Male T= 8/8, C= 14/14Female T= 0/8, C= 0/14**Value (Mean ± SD)**Age (years): T= 64 ± 5, C= 61 ± 6Body Mass Index (kg/m^2^): T= 40.6 ± 4.2, C= 38.6 ± 2.8Creatinine level (mg/dL): T= 1.2 ± 0.2, C= 1.1 ± 0.2Albumin level (g/dL): T= 3.8 ± 0.4, C= 3.6 ± 0.2Ejection fraction (%): T= 50 ± 10, C= 51 ± 8**Comorbidities**Diabetes mellitus: T= 6/8, C= 8/14Peripheral vascular disease: T= 0/8, C= 1/14Hypertension: T= 8/8, C= 14/14Cerebral vascular disease: T= 1/8, C= 3/14COPD: T= 4/8, C= 2/14Current smoker: T= 2/8, C= 2/14**Intraoperative variables**Isolated CABG: T= 3/8, C= 13/14Isolated AVR: T= 1/8, C= 1/14	Rigid sternal fixation	Wire fixation	**Value (Mean ± SD)**Cardiopulmonary bypass time (minutes): T= 145 ± 57, C= 898 ± 41Duration of procedure (hours): T= 6.03 ± 1.36, C= 6.88 ± 1Duration of hospital stay (days): T= 17 ± 5, C= 11 ± 3Morphine usage (mg/hour): T= 1.3 ± 1.2, C= 3.6 ± 2.3**Complication**Sternal dehiscence: T= 0, C= 1/14
7	9	Matsuyama *et al*, 2016 ^[17]^ Retrospective study	N= 64 (T= 31, C= 33)Male T= 29/31, C= 26/33Female T= 2/31, C= 7/33**Value (Mean ± SD)**Age (years): T= 65.1 ± 7.1, C= 70.5 ± 10.6Body Mass Index (kg/m^2^): T= 23.3 ± 3.2, C= 24.0 ± 3.8Body Surface Area (m^2^): T= 1.69 ± 0.16, C= 1.65 ± 0.17**Comorbidities**Diabetes mellitus: T= 22/31, C= 16/33Insulin dependent: T= 20/31, C= 3/33Hypertension: T= 28/31, C= 31/33Hyperlipidaemia: T= 24/31, C= 25/33Undergoing haemodialysis: T= 7/31, C= 3/33Unstable angina: T= 7/31, C= 8/33Smoker: T= 19/31, C= 24/33	Rigid sternal fixation	Wire fixation	**Value (Mean ± SD)**Duration of procedure (hours): T= 6.28 ± 1.28, C= 5.9 ± 1.3Duration of hospital stay (days): T= 16.0 ± 4.0, C= 19.3 ± 5.4Blood transfusion: T= 19/31, C= 20/33**Pain scores (0 representing no pain and 10 representing the worst pain)**At 1-4 POD: T= 2.1 ± 0.5, C= 2.3 ± 0.5At 5-8 POD: T= 0.9 ± 0.6, C= 1.4 ± 0.6At 9-12 POD: T= 0.2 ± 0.3, C= 1.0 ± 0.5**Analgesic usage count**At 1-4 POD: T= 1.6 ± 1.6, C= 1.7 ± 1.9At 5-8 POD: T= 0.7 ± 1.2, C= 1.7 ± 2.2At 9-12 POD: T= 0.1 ± 0.5, C= 0.8 ± 1.5
8	10	Peigh *et al*, 2017 ^[18]^ Randomized controlled trial	N= 80 (T= 39, C= 41)Male T= 31/39, C= 34/41Female T= 8/39, C= 7/41Age (years) (Mean ± SD: T= 65 ± 8, C= 66 ± 9**Comorbidities**Myocardial infarction: T= 10/39, C= 5/41Congestive heart failure: T= 1/39, C= 4/41Reduced ejection fraction: T= 4/39, C= 4/41Chronic atrial fibrillation: T= 2/39, C= 0Left main disease: T= 27/39, C= 24/41Hypertension: T= 33/39, C= 33/41Diabetes: T= 17/39, C= 15/41Hyperlipidaemia: T= 25/39, C= 29/41History of stroke: T= 1/39, C= 1/41Peripheral vascular disease: T= 2/39, C= 2/41Chronic lung disease: T= 4/39, C= 4/41**Intraoperative variables**Isolated coronary artery bypass grafting: T= 27/39, C= 29/41Valve only: T= 10/39, C= 8/41Coronary artery bypass grafting and valve: T= 2/39, C= 4/41	Rigid sternal fixation	Wire fixation	**Value (Mean ± SD)**Duration of ventilator use (hour): T= 7.3 ± 4.8, C= 9.4 ± 8.7Duration of intensive care unit stay (hour): T= 51 ± 29, C= 55 ± 44Duration of hospital stay (days): T= 6.8 ± 4.4, C= 8.0 ± 6.9**Pain scores (0 representing no pain and 10 representing the worst pain)**At POD 1: T= 2.3 ± 3.1, C= 2.8 ± 3.4At POD 2: T= 2.1 ± 2.7, C= 2.0 ± 2.7At POD 3: T= 0.5 ± 1.4, C= 0.8 ± 1.7At POD 4: T= 0.5 ± 1.8, C= 1.7 ± 3.4At POD 5: T= 0.2 ± 1.0, C= 0.4 ± 1.3**Analgesic requirement**At POD 1: T= 45.8 ± 125.8, C= 45.8 ± 158.4At POD 2: T= 21.2 ± 66.0, C= 14.7 ± 34.1At POD 3: T= 6.1 ± 6.2, C= 9.0 ± 16.6At POD 4: T= 3.7 ± 5.2, C= 16.2 ± 66.5At POD 5: T= 3.3 ± 5.5, C= 8.8 ± 23.7Total narcotic use: T= 79.5 ± 141.4, C= 87.7 ± 256.7**Complications**Pneumonia: T= 0, C= 2/41Postoperative stroke: T= 1/39, C= 1/41Need for haemodialysis: T= 0, C= 1/41New onset atrial fibrillation: T= 9/39, C= 14/41Total sternal infection: T= 2/39, C= 3/41Superficial sternal infection: T= 1/39, C= 3/41Deep sternal infection: T= 1/39, C= 0Total Mortality: T= 0, C= 1/41
9	11	Qiu *et al*, 2022 ^[19]^ Retrospective study	N= 689 (T= 411, C= 278)Male T= 277/411, C= 173/278Female T= 134/411, C= 105/278**Value (Mean ± SD)**Age (years): T= 61.7 ± 10.6, C= 61.1 ± 12.4Body mass index (kg/m^2^): T= 24.6 ± 3.5, C= 23.9 ± 3.6**Intraoperative variables**Coronary artery bypass grafting (CABG): T= 218/411, C= 133/278Valvular: T= 116/411, C= 98/278CABG + valvular: T= 22/411, C= 17/278Congenital heart disease: T= 11/411, C= 11/278Aortic: T= 28/411, C= 13/278Others: T= 16/411, C= 6/278On-pump surgery: T= 289/411, C= 178/278**Comorbidities**Hypertension: T= 211/411, C= 150/278Type 2 diabetes: T= 99/411, C= 60/278	Rigid sternal fixation	Wire fixation	**Value (Mean ± SD)**Duration of Intensive care unit stay (hour): T= 93.4 ± 75.6, C= 81.3 ± 55.2Duration of hospital stay (days): T= 24.45 ± 26.8, C= 27.9 ± 24.9**Drain volume**First 24 hours (milliliter): T= 361.5 ± 242.6, C= 469.7 ± 337.4Total volume (milliliter): T= 692.4 ± 395.6, C= 840.3 ± 477.2**Complications**Mortality in 30 days: T= 22/411, C= 15/278Total sternal complications: T= 11/411, C= 14/278
10	12	Raman *et al*, 2012 ^[20]^ Randomized controlled trial	N= 140 (T= 70, C= 70)Male T= 51/70, C= 52/70Female T= 19/70, C= 18/70**Value (Mean ± SD)**Age (years): T= 66.3 ± 9.8, C= 64.0 ± 8.9Body mass index (kg/m^2^): T= 31.8 ± 5.5, C= 31.8 ± 4.6**Comorbidities**Diabetes mellitus: T= 48/70, C= 43/70Renal failure: T= 19/70, C= 19/70COPD: T= 15/70, C= 19/70Osteoporosis: T= 4/70, C= 6/70	Rigid sternal fixation	Wire fixation	**Value (Mean ± SD)** **At 3 months**Sternal healing score: T= 1.7 ± 1.1, C= 0.9 ± 0.8**At 6 months**Sternal healing score: T= 3.2 ± 1.6, C= 2.2 ± 1.1**Pain score**(0 representing no pain and 10 representing the worst pain)1 week: T= 3.1 ± 1.8, C= 3.6 ± 1.93 weeks: T= 2.3 ± 1.2, C= 3.1 ± 1.86 weeks: T= 2.3 ± 1.7, C= 2.7 ± 1.93 months: T= 1.9 ± 1.1, C= 2.0 ± 1.36 months: T= 1.9 ± 1.2, C= 1.9 ± 1.3
11	13	Royse *et al*, 2020 ^[21]^ Randomized controlled trial	N= 50 (T= 26, C= 24)Male T= 21/26, C= 17/24Female T= 5/26, C= 7/24**Value (Mean ± SD)**Age (years): T= 64.2 ± 14.7, C= 64.8 ± 13.9BMI (kg/m^2^): T= 28.7 ± 4.7, C= 27.6 ± 4.3**Comorbidities**Diabetes mellitus: T= 11/26, C= 6/24COPD: T= 6/26, C= 3/24Hypertension T= 18/26, C= 15/24Osteoporosis: T= 1/26, C= 1/24Smoker: T= 2/26, C= 1/24Ex-smoker: T= 12/26, C= 12/24**Intraoperative variables**CABG: T= 18/26, C= 19/24Valve surgery: T= 2/26, C= 3/24CABG + valve: T= 6/26, C= 2/24	Rigid sternal fixation	Wire fixation	**Value (Mean ± SD)**Cardiopulmonary bypass time (minutes): T= 117.6 ± 58, C= 111.5 ± 41.8Sternal closure time (minutes): T= 16.2 ± 6.7, C= 12.6 ± 5.8Duration of intensive care unit stay (hours): T= 60 ± 108, C= 43.2 ± 24Duration of hospital stay (days): T= 9.1 ± 3.1, C= 9 ± 4.5**At 6 weeks**Sternal edge movement of >2mm at >2 sites: T= 1/25, C= 7/22**At 12 weeks**Sternal edge movement of >2mm at >2 sites: T= 0, C= 5/20
12	14	Synder *et al*, 2009 ^[22]^ Retrospective study	N= 129 (T= 30, C= 99)Male T= 30/30, C= 98/99Female T= 0, C= 1/99**Value [Median (IQR)]**Age (years): T= 61 (43-78), C= 59 (44-77)BMI (kg/m^2^): T= 33 (22-47), C= 32 (25-49)**Comorbidities**Current smoker: T= 13/30, C= 20/99Diabetes mellitus: T= 3/30, C= 18/99LVEF <35%: T= 4/30, C= 12/99Obesity: T= 22/30, C= 84/99**Intraoperative variables**Isolated CABG: T= 24/30, C= 79/99Isolated valve replacement: T= 1/30, C= 12/99CABG + valve: T= 5/30, C= 8/99	Rigid sternal fixation	Wire fixation	**Value (Mean ± SD)**Cardiopulmonary bypass time (minutes): T= 85 ± 25, C= 89 ± 25.67Duration of procedure (hours): T= 4.3 ± 1.01, C= 4.05 ± 0.45Duration of hospital stay (days): T= 7 ± 7.5, C= 8 ± 32.83**Complications**Total sternal complications: T= 3/30, C= 22/99Sternal complications at 30 days: T= 0/30, C= 12/99Sternal complications after 30 days: T= 3/30, C= 10/99
13	15	Tamura *et al*, 2023 ^[23]^ Retrospective study	N= 116 (T= 47, C= 69)Male T= 33/47, C= 48/69Female T= 14/47, C= 21/69**Value (Mean ± SD)**Age (years): T= 69.3 ± 8.4, C= 70.8 ± 10.4BMI (kg/m^2^): T= 24.3 ± 4.2, C= 23.3 ± 3.6**Comorbidities**Smoker: T= 5/47, C= 10/69Hypertension: T= 45/47, C= 60/69Diabetes mellitus: T= 36/47, C= 53/69Dyslipidaemia: T= 40/47, C= 57/69Peripheral arterial disease: T= 7/47, C= 10/69Chronic kidney disease: T= 22/47, C= 37/69	Rigid sternal fixation	Wire fixation	**Value (Mean ± SD)**Duration of intensive care unit stay (hours): T= 96 ± 28.8, C= 117.6 ± 21.6Duration of hospital stay (days): T= 15.2 ± 3.8, C= 18.0 ± 5.0Maximum Prince Henry Pain scale (0 is no pain on cough and 4 is severe pain at rest): T= 1.6 ± 1.7, C= 2.7 ± 1.4**Complications**Re-exploration: T= 0, C= 3/69Surgical site infection: T= 0, C= 3/69Mortality: T= 1/47, C= 3/69
14	16	Tugulan *et al*, 2020 ^[24]^ Retrospective study Obese patient population	N= 184 (T= 58, C= 126)Male T= 41/58, C= 77/126Female T= 17/58, C= 49/126**Value (Mean ± SD)**Age (years): T= 56.8, C= 58.6BMI (kg/m^2^): T= 40.2, C= 39.5**Comorbidities**Tobacco use: T= 32/58, C= 75/126Diabetes mellitus: T= 37/58, C= 67/126COPD: T= 6/58, C= 19/126**Intraoperative variables**CABG: T= 27/58, C= 62/126Aortic valve replacement: T= 18/58, C= 22/126Left ventricular assist device placement: T= 2/58, C= 25/126Mitral valve repair/replacement: T= 8/58, C= 14/126Ascending aorta or root replacement: T= 6/58, C= 14/126	Rigid sternal fixation	Wire fixation	**Value (Mean ± SD)**Duration of hospital stay (days): T= 8 ± 8.75, C= 12 ± 16.33Duration of intensive care unit stay (hours): T= 96 ± 132, C= 144 ± 355.92Duration of ventilator use (hours): T= 37.9, C= 48.8**Complications**Sternal complications: T= 3/58, C= 6/126Total Mortality: T= 3/58, C= 6/126

### Quantitative synthesis

#### Sternal healing score

##### Sternal healing score at 3 months

**Figure 2.**
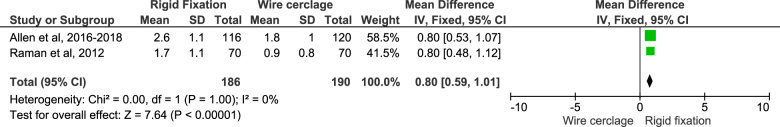
Sternal healing score at 3 months.

Data of sternal healing score at 3 months was pooled from two studies using the fixed effect model and the difference in the sternal healing was found to be statistically significant. The rigid fixation group had better healing score at 3 months (MD: 0.8; 95% CI: 0.59–1.01; *n* = 376; *I*^2^ = 0%; *P* = < 0.00001) compared to the wire closure (Fig. 2).

##### Sternal healing score at 6 months

**Figure 3.**
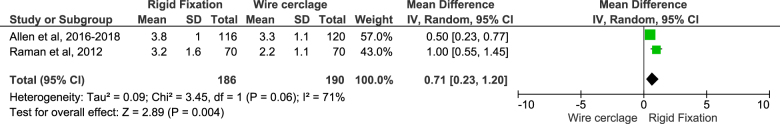
Sternal healing score at 6 months.

Sternal healing score at 6 months was reported by two studies and pooling the data using the random effect model showed statistically significant difference in the healing score. The rigid fixation group had better healing score at 6 months (MD: 0.71; 95% CI: 0.23–1.20; *n* = 376; *I*^2^ = 71%; *P* = 0.004) compared to the wire closure (Fig. 3).

#### Duration outcomes

##### Duration of sternal closure

**Figure 4.**
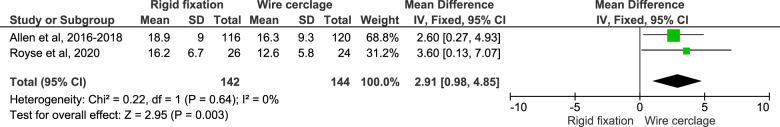
Duration of sternal closure.

Two studies reported the duration of sternal closure outcome and on pooling the data using the fixed effect model, it was observed that the wire cerclage group took an average of 2.91 minutes lesser for sternal closure (MD: 2.91; 95% CI: 0.98–4.85; *n* = 286; *I*^2^ = 0%; *P* = 0.003) (Fig. 4).

##### Duration of hospital stay

**Figure 5.**
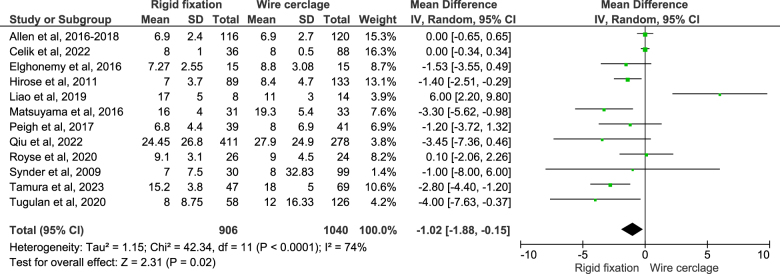
Duration of hospital stay.

**Figure 6.**
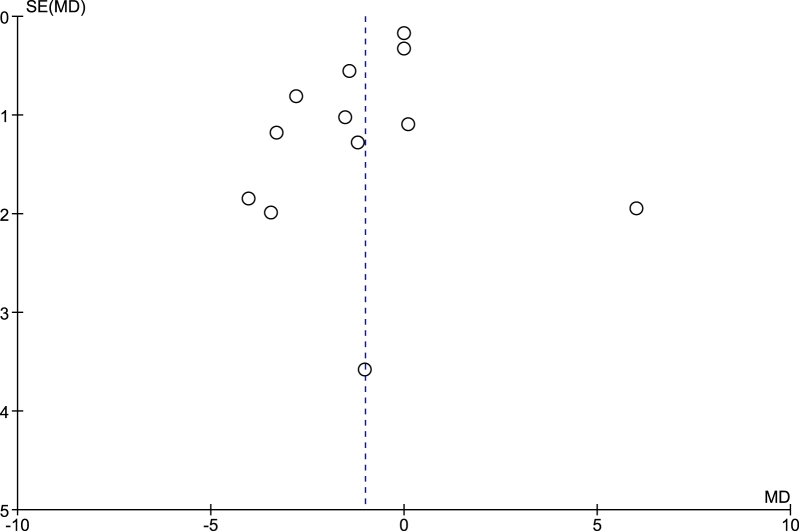
Funnel plot for duration of hospital stay outcome.

A total of 12 studies reported the duration of hospital stay outcome and on pooling the data using the random effect model, it was found that the rigid fixation group had 1.02 days lesser hospital stay in an average (MD: −1.02; 95% CI: −1.88–(−0.15); *n* = 1946; *I*^2^ = 74%; *P* = 0.02) (Fig. 5). On sensitivity analysis, no significant changes were seen in the obtained result. On assessing the publication bias through funnel plot, the plot was seen to be asymmetrical (Fig. 6).

On sub-group analysis of randomized studies only, no statistically significant difference was observed. Three randomized studies reported the duration of hospital stay outcome and pooling the data showed no difference between two groups (Figure C, Supplementary File 2 http://links.lww.com/MS9/A788). On sub-group analysis of studies that included obese population only, no statistically significant difference was observed. Three studies with obese population had reported the duration of hospital stay outcome and pooling the data showed no difference between two groups (Figure D, Supplementary File 2 http://links.lww.com/MS9/A788).

##### Duration of intensive care unit (ICU) stay

On pooling the data of duration of ICU stay from seven studies by using the random effect model, it did not show statistically significant changes (MD: −8.33; 95% CI: −21.12–4.47; *n* = 1465; *I*^2^ = 85%; *P* = 0.20) (Figure E, Supplementary File 2 http://links.lww.com/MS9/A788). No changes were observed in the sensitivity analysis, sub-group analysis with obese population only, and in the sub-group analysis of randomized studies.

##### Duration of ventilator use

Duration of ventilator outcome was reported by four studies and pooling the data by using the random effect model, it did not show any statistically significant result (MD: −0.87; 95% CI: −3.51–1.78; *n* = 456; *I*^2^ = 75%; *P* = 0.52) (Figure F, Supplementary File 2 http://links.lww.com/MS9/A788). No changes were observed in the sensitivity analysis.

#### Pain scores

##### Pain score within first week of operation

**Figure 7.**
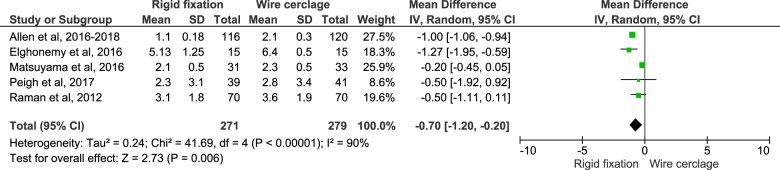
Pain score within first week of operation.

On pooling data from five studies by using random effect model, it was found that the rigid fixation group had experienced lesser pain and the difference in the pain score was statistically significant (MD: −0.70; 95% CI: −1.20–(−0.20); *n* = 550; *I*^2^ = 90%; *P* = 0.006) (Fig. 7). Sensitivity analysis did not show any changes in these figures. On sub-group analysis of randomized studies, it was observed that the rigid fixation group had experienced lesser pain and the difference in the pain score was statistically significant (MD: −0.99; 95% CI: −1.06–(−0.93); *n* = 456; *I*^2^ = 33%; *P* = < 0.00001).

##### Pain score at 3 months of operation

**Figure 8.**
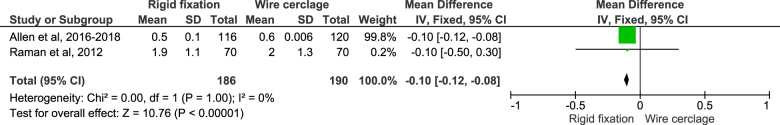
Pain score at 3 months of operation.

Two studies reported the pain scores at 3 months and on pooling the data by using the fixed effect model, it was found that the rigid fixation group had experienced lesser degree of pain and this difference was statistically significant (MD: −0.10; 95% CI: −0.12–(−0.08); *n* = 376; *I*^2^ = 0%; *P* = < 0.00001) (Fig. 8).

##### Pain score at 6 months of operation

**Figure 9.**
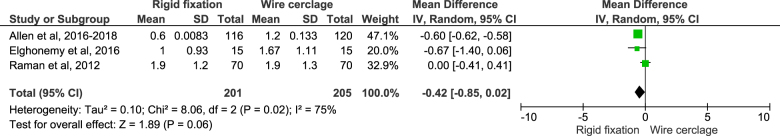
Pain score at 6 months of operation.

Three studies reported the pain scores at 6 months of operation and on pooling the data by using the random effect model, it was found that the difference in pain score between the two groups was not statistically significant (MD: −0.42; 95% CI: −0.85–0.02; *n* = 406; *I*^2^ = 75%; *P* = 0.06) (Fig. 9). On sensitivity analysis, on excluding one study (Raman *et al*, 2012), the pain score was seen to be better in rigid fixation group and the difference was statistically significant (MD: −0.60; 95% CI: −0.62–(−0.58); *n* = 266; *I*^2^ = 0%; *P* = < 0.00001).

#### Complications

A comparison of various complications like total mortality, sternal infection, sternal dehiscence, and need for re-exploration was done by using the odds ratio as effect measure, and among the analyses, sternal dehiscence was found to be significantly more in the wire closure group, while total mortality, sternal infection, and need for re-exploration outcomes were found to have no significant difference. Sensitivity analysis and subgroup analysis were also performed for these outcomes. The details of the analysis are given in Table 2.

**Table 2:** Analysis of various complications

Outcomes	Statistics	Included studies	Effect model	Sensitivity/Sub-group analysis
Total Mortality	OR: 1.03; 95% CI: 0.38-1.19; *I*^2^ = 0%; *P* = 0.89	8	Fixed	No significant changes in the result in sensitivity analysis. No statistically significant difference in sub-group analysis of randomized studies and obese patient population.
Sternal infection	OR: 0.64; 95% CI: 0.24-1.69; *I*^2^ = 19%; *P* = 0.37	4	Fixed	No significant changes in the result in sensitivity analysis. No statistically significant difference in sub-group analysis of randomized studies.
Sternal dehiscence	OR: 0.23; 95% CI: 0.08-0.66; *I*^2^ = 0%; *P* = 0.006	3	Fixed	The result obtained was seen to statistically insignificant when one study (Atik *et al*, 2023) was removed. Sternal dehiscence outcome was reported by the studies that included obese population only. So, the sub-group analysis for obese population is as same as the main analysis.
Re-exploration	OR: 0.49; 95% CI: 0.11-2.12; *I*^2^ = 23%; *P* = 0.34	3	Fixed	No significant changes in the result in sensitivity analysis.

## Discussion

Median sternotomy continues to be the standard approach for accessing the mediastinum in cardiac surgery^[3,10]^. However, postoperative complications such as sternal instability, dehiscence, wound infection, and subsequent mediastinitis remain significant concerns, contributing to substantial postoperative morbidity and mortality. Therefore, employing techniques that ensure stable sternal approximation is crucial for minimizing these complications^[3]^. We conducted a systematic analysis comparing the effectiveness of two sternal approximation techniques: rigid plate fixation and standard wire closure for post-sternotomy closure. Rigid fixation demonstrated significantly improved sternal wound healing at both 3 and 6 months, reduced pain scores at 1 month, and shortened hospital stays. Conversely, wire closure had the advantage of a shorter sternal closure duration but was associated with a higher incidence of sternal dehiscence. However, our study found no significant differences between the two techniques in terms of ICU length of stay, duration of ventilator use, pain at 6 months, overall mortality, sternal infection rates, or the need for re-exploration.

Bone healing follows biomechanical principles, emphasizing the precise approximation of the osteotomy gap to achieve effective osteosynthesis aided by biological factors in the absence of risk factors^[11,12]^. This process ensures stability through cohesive forces acting on the fused bone^[13]^. Similarly, surgical sternotomy wound healing aligns with these principles, where proper alignment and fusion are essential for maintaining sternal stability^[12]^. Effective sternal coaptation not only enhances stability but also accelerates recovery by improving bone healing rates and reducing pain, and also decreases the risk of sternal dehiscence, infection, and the need for reoperation. Moreover, the maintenance of sternal stability depends on factors such as sternal quality, the presence of risk factors, and the chosen closure techniques^[4]^. In this review, rigid fixation techniques have demonstrated superior outcomes in promoting sternal healing over traditional wire cerclage. By providing greater biomechanical stability, rigid fixation minimizes micromotion at the sternal edges, facilitating early bone regeneration and effective lateral approximation^[3]^. This is reflected by the result of this study that has favored the rigid fixation group in the duration of hospital stay outcome and incidence of sternal dehiscence.

One of the highlights of this study is its finding of a significant difference in the incidence of sternal dehiscence among two groups. The population included in these analyses was from the obese group only, and it can be said that rigid fixation is superior in preventing sternal dehiscence among obese patients after median sternotomy. It has been identified that the sternal dehiscence is a real problem when BMI is more than 30 and is associated with high morbidity^[14]^. Literature also suggests obesity as the significant independent predictor of adverse outcomes after coronary artery bypass operation and is associated with deep sternal wound infection^[15]^. Among the obese population, in the concurrent presence of sternal infection, sternal dehiscence is thought to occur when the sternal wire cuts through the bone, causing multiple fractures in the sternum^[14]^. In this study, the use of rigid fixation has been found to decrease this complication among the obese population. This also contributes to decreasing the duration of hospital stay.

Post-sternotomy pain is a critical factor influencing enhanced recovery after surgery (ERAS) leading to early rehabilitation and quality of life^[16,17]^. Our analysis demonstrates that rigid fixation is associated with significantly lower pain scores in the first and the third post-sternotomy months. This benefit can be attributed to the improved sternal stability provided by rigid fixation, which minimizes stress on periosteal nerves and the surrounding soft tissues. Additionally, rigid fixation promotes primary bone healing without callus, leading to early stability and recovery^[18]^. In contrast, wire closure techniques are associated with higher postoperative pain requiring high analgesics, which may be exacerbated by micromotion of the sternal edges, eversion of twisted wires, wire-tissue friction, and wire fractures. These findings highlight the advantages of rigid fixation in reducing postoperative discomfort across diverse patient populations^[16]^. However, the absence of significant differences in pain scores between the two techniques at 6 months likely reflects the natural timeline of secondary bone healing, as the transition from inflammation to the remodeling phase can extend for months to years^[19]^.

This study has included data from different studies, and out of the included studies, the majority of them were non-randomized retrospective studies. This has increased the uncertainty of having either an overestimation or an underestimation of the obtained results. The inclusion criteria of the different included studies were different, and this also has contributed to the heterogeneity. Although the sub-group analysis was performed, some analyses had high heterogeneity, which may be attributed to the non-random sampling of the participants and differences in the execution of surgical procedures by different surgeons, difference in the post-operative care, differences in the inclusion criteria of individual studies, and differences in the study setting as a whole. The duration of the hospital stay outcome favoured the rigid fixation group; however, the difference was marginal, and it also suggested publication bias. This outcome needs to be evaluated further through randomized studies. Also, we could not compare the cost of care between these two methods due to a lack of data. For the consolidation of the results obtained, more randomized trials that have a focus on postoperative pain, duration of ventilator use, duration of hospital stay, duration of ICU stay, and cost of care are needed.

## Conclusion

Rigid fixation was found to be superior to the wire cerclage in regard to shorter hospital stay duration, sternal healing scores, postoperative pain up to 3 months, and sternal dehiscence complication among the obese population. As this study had its limitations, further randomized trials with a focus on the duration of ventilator use, duration of hospital stay, duration of ICU stay, and cost of care are needed.

## Data Availability

Analyzed data is publicly available (is present within the manuscript).

## Research registration unique identifying number (UIN)

Protocol followed in this study was registered in the Registry of Systematic Reviews/Meta-Analyses (Unique Identifying Number: reviewregistry1938); URL: https://researchregistry.knack.com/researchregistry#registryofsystematicreviewsmeta-analyses/registryofsystematicreviewsmetaanalysesdetails/677548c30cefc902ffedbf45/. Protocol followed in this study was prospectively registered in the International prospective register of systematic reviews (PROSPERO ID: CRD42024590743); URL: https://www.crd.york.ac.uk/prospero/display_record.php?ID=CRD42024590743.
